# Burden and associated factors of anemia among pregnant women attending antenatal care in southern Ethiopia: cross sectional study

**DOI:** 10.1186/s13104-017-2605-x

**Published:** 2017-07-14

**Authors:** Weinshet Getahun, Tefera Belachew, Amare Desalegn Wolide

**Affiliations:** 1 Department of Biomedical Sciences, College of Health Sciences, Jimma University, Jimma, Ethiopia; 2 Department of Reproductive and Population Health, College of Health Sciences, Jimma University, Jimma, Ethiopia

**Keywords:** Anemia, Pregnancy, Antenatal care

## Abstract

**Background:**

Anemia is a condition in which the number of red blood cells or their oxygen-carrying capacity is insufficient to meet physiologic needs, which varies by age, sex, altitude, smoking, and pregnancy status. The study aim is to determine the prevalence and factors associated with anemia among pregnant women attending a hospital in southern Ethiopia using a structured interview administered questionnaire.

**Methods:**

Facility-based cross-sectional study was conducted from March 01–April 30 2015 at Butajira General Hospital, Ethiopia. A total of 217 women responded to the questionnaire and provided blood and stool samples for analysis. Data were analyzed using Statistical packages for social sciences version 20 for windows.

**Result and conclusions:**

The overall burden of anemia in this study was 27.6%. Residence, ANC follow up, history of excess menstrual bleeding and interpregnancy interval were statistically associated with anemia among the pregnant women. Therefore, working in the identified gaps could reduce the current burden of anemia among pregnant women in the study area.

## Background

Anemia is a condition in which the number of red blood cells or their oxygen-carrying capacity is insufficient to meet physiologic needs, which varies by age, sex, altitude, smoking, and pregnancy status [1]. Anemia is a global public health problem affecting both developing and developed countries with major consequences for human health as well as socio-economic development. It occurs at all stages of the life cycle but is more prevalent in pregnant women and young children [2]. In developing countries, the cause of anemia during pregnancy is multifactorial and includes nutritional deficiencies of iron, folate, and vitamin B_12_ and parasitic diseases, such as malaria and hookworm. The relative contribution of each of these factors to anemia during pregnancy varies greatly by geographical location, season, and dietary practice [3]. During pregnancy, anemia is a major cause of morbidity and mortality of pregnant women in developing countries and has both maternal and fetal consequences [4–9]. It may also lead to premature births [10], low birth weight [11], fetal cognitive impairment, and death [12, 13]. According to the report of World Health Organization (WHO), the prevalence of anemia among pregnant women is 41.8%. The highest prevalence rate (61.3%) is found among pregnant women in Africa [14]. According to the Ethiopian Demographic Health Survey (EDHS) report, 17% women are estimated to be anemic and 22% of the pregnant women are anemic [15]. Because of different Socio-economic status, behavioral, geographical and methodological difference (design and methods) the national figure of EDHS could not represent the prevalence of anemia among pregnant women found in different part of Ethiopia. Thus, disclosing the burden and associated factor of anemia among pregnant women in the study area will help to develop evidence-based decision and intervention strategies to improve the health status of pregnant women. Furthermore, it will contribute to discount predisposing factors of anemia. Therefore, this study is aimed to determine the burden of anemia and associated risk factors among pregnant women attending Antenatal Care Clinic (ANC) follow up at Butajira General Hospital, Southern Ethiopia.

## Methods

### Study setting and design

Facility based cross-sectional study was done at Butajira General Hospital from March 01 to April 30, 2015 Southern Ethiopia. Butajira General Hospital is located in the South Nation Nationality and Peoples Region (SNNPR), which is 135 km far from Addis Ababa, the capital city of Ethiopia. On average, 405 pregnant women have been registered in the ANC follow up.

### Study population

All pregnant women visiting ANC follow up from March 01 to April 30, 2015 and fulfilled the inclusion criteria included to the study. Current illness and drug treatment for chronic illness were the exclusion criteria. Subject recruitment was conducted on volunteer basis after having a clear understanding of the research.

### Sample size determination and sampling procedures

The sample size was determined based on the single population proportion formula using Z^2^ × p × q/d^2^ with a 95% confidence interval, 5% margin of error and an assumption that 50% of pregnant women are anemic in the study area. Thus, the total sample size was 384.16. Since the total ANC followers in the study area are 405 which is less than 10,000 we used correction formula to calculate the final sample size. Therefore, considering non-response rate (10%), the final sample size for this study were 217. The sample random sampling (lottery) method was used to select the first study participant and a systematic random sampling method (k = N/n = 405/217 = 1.86 = 2) was used to select the rest of the others. Thus, every second (k = 2) pregnant women who mate our inclusion criteria was selected.

### Methods of data collection

Information on socio-demographic characteristics, obstetric and gynecological history, and dietary patterns were collected using interviewer-administered pretested questionnaires. Hematological parameters such as Mean cell volume (MCV), Mean cell hemoglobin (MCH) and Mean cell hemoglobin concentration (MCHC) and the analysis were done using CELL-DYN 1800. Anemia in pregnancy was defined as Hemoglobin (Hb) less than 11 g/dl [16]. Stool specimens were collected from each study participant and an examination of intestinal parasitic infections was conducted using saline wet smear and formol-ether concentration techniques [17]. Bivariate and multivariate logistic regression analysis were done to assess the independent risk factors associated with anemia. The goodness of fit of the final logistic model was tested using Hosmer and Lemeshow test (p < 0.05).

### Data quality assurance

To assure the quality of the data Standard operating procedures (SOPs) were followed during specimen collection and other laboratory procedures. All reagents used were checked for their expiry date and prepared according to the manufacturer’s instructions. Training was given for the data collectors to minimize technical and observer biases.

### Methods of data analysis

Data were edited, cleaned and checked for its completeness and entered into EpiData 3.1 then exported to Statistical packages for social sciences (SPSS) Version 20 for analysis. Categorical variables were summarized as numbers and percentages. Both bivariate and multivariate logistic regression analysis were done to assess the independent risk associated factors for anemia. All variables with a p value less than 0.05 were considered as statistical significance. The goodness of fit of the final logistic model was tested using Hosmer and Lemeshow test at a p value less than 0.05.

### Ethical statement

Ethical clearance was obtained from Jimma University Ethical Review Committee. Letter of permission to conduct the study was obtained from Butajira General Hospital ANC clinic. Written informed consent was obtained from each study participant. The purpose of the study was clearly described to the study participants including the benefits and risks of the study. Any information concerning the participants was kept confidential and the specimen collected from the participants was only analyzed for the intended purposes. The participants were enrolled only after they were sufficiently counseled and their informed consents have been obtained. Positive findings for intestinal helminths and hemoglobin value below accepted value were communicated with the respective health professionals of Butajira General Hospital ANC clinic for possible interventions.

## Results

### Socio-demographic characteristics

A total of 217 study participants were involved in the study. The mean (±SD) age of the study participants were 26.87 (±5.703). The majority of the study subjects, 213 (98.1%) were married, 94 (43.3%) were primary school completed, 99 (45.6%) where housewife and 168 (77.4%) were urban inhabitants (Table 1).

**Table 1:** Bivariate analysis of socio-demographic characteristics and anemia among pregnant women (n = 217) in Butajira General Hospital, Southern Ethiopia, March 1–April 30, 2015

Variables	Total (n = 217) n (%)	Anaemia		COR (95% CI)	p value
		Yes (n = 60) n = (%)	No (n = 157) n = (%)		
Age in years					
<20	17 (7.8)	7 (3.2)	10 (4.6)	1r	0.755
21–30	94 (43.3)	29 (48.3)	65 (41.4)	2.105 (0.210–1.621)	
31–40	70 (32.2)	18 (30)	52 (33.1)	0.606 (0.166–1.540)	
>40	36 (16.5)	6 (10)	30 (19.1)	1.650 (0.208–4.882)	
Educational status					
Illiterate	32 (14.7)	11 (5.1)	21 (9.6)	1r	0.999
Primary	94 (43.3)	30 (13.8)	64 (29.4)	0.895 (0.383–2.091)	
Secondary	72 (33.2)	15 (6.9)	57 (26.2)	0.502 (0.199-1.267)	
Tertiary	15 (6.9)	4 (1.8)	11 (5.1)	0.694 (0.179–2.697)	
Others	4 (1.8)	0 (0)	4 (1.8)	0.000 (0.000, –)	
Occupation status					
Housewife	99 (45.6)	28 (12.9)	71 (32.7)	1r	0.931
Trader	61 (28.1)	15 (6.9)	46 (21.1)	0.827 (0.399–1.713)	
Daily laborer	11 (5.1)	4 (1.8)	7 (3.2)	1.449 (0.393–5.338)	
Farming	6 (2.8)	3 (1.3)	3 (1.3)	2.536 (0.483–13.323)	
Self employed	10 (4.6)	2 (0.9)	8 (3.68)	0.634 (0.127–3.172)	
NGO employee	6 (2.8)	1 (0.46)	5 (2.3)	0.507 (0.057–4.537)	
Governmental employee	24 (11.1)	7 (3.2)	17 (7.8)	1.044 (0.391–2.790)	
Residence					
Urban	168 (77.4)	40 (18.4)	128 (58.9)	0.453 (0.232–0.887)	0.021*
Rural	49 (22.6)	20 (9.2)	29 (13.3)	1r	
Marital status					
Single	4 (1.8)	4 (1.8)	0 (0)	1r	0.999
Married	213 (98.2)	56 (25.8)	157 (72.3)	–	
Monthly income (ETB)					
<500	80 (36.9)	24 (11)	56 (25.8)	1r	0.453
500–1000	79 (36.4)	22 (10.1)	57 (26.2)	0.901 (0.453–1.788)	
>1000	58 (26.7)	14 (6.4)	44 (20.2)	0.742 (0.344–1.601)	

*1r* set as reference, *NGO* non-governmental employee, *1 US $* 18.6 Ethiopian Birr (ETB)

* p < 0.05

### Health care related factors

From the total 217 study participants, 54 (24.9%) and 53 (24.4%) study subjects had the history of malarial infection prior to the data collection time and excess menstrual related bleeding respectively. The majority of the study participants, 171 (78.8%) had ANC follow-up and 105 (48.4%) had a habit of eating iron-rich animal foods sources (AFS) such as red meat, milk and milk products, egg, poultry, and fish. Study participants with a birth interval of fewer than 2 years 46 (76.7%) had shown to be more anemic than those with an interval of greater than or equal to 2 years 5 (8.3%). Among the study participants, 28 (13%) were infected with intestinal parasites and 21 (9.6%) of them were anemic. Blood film examination was also done to identify blood hemoparasites, particularly for malaria. However, no hemoparasite was identified during the study period (Table 2).

**Table 2:** Bivariate analysis of factors associated with anaemia among pregnant women (n = 217) in Butajira General Hospital, Southern Ethiopia, March 1–April 30, 2015

Variables	Total (n = 217) n (%)	Anaemia		COR (95% CI)	p value
		Yes (n = 60) n = (%)	No (n = 157) n = (%)		
History of Malaria					
Yes	54 (24.9)	24 (40)	30 (19.1)	1r	0.002*
No	163 (75.1)	36 (60)	127 (80.9)	0.354 (0.185–0.680)	
History of excess menstrual bleeding					
Yes	53 (24.4)	44 (73.3)	9 (5.7)	1r	<0.0001*
No	164 (75.6)	16 (26.7)	148 (94.3)	0.022 (0.009–0.053)	
Bare foot walking					
Yes	19 (8.8)	7 (11.7)	12 (7.5)	1r	0.352
No	198 (91.2)	53 (88.3)	145 (92.5)	0.627 (0.234–1.676)	
ANC follow up					
Yes	171 (78.8)	24 (40)	147 (93.6)	0.045 (0.020–0.103)	<*0.0001* *
No	46 (21.2)	36 (60)	10 (6.3)	1r	
ITN utilization					
Yes	81 (37.3)	17 (28.3)	64 (40.8)	0.574 (0.301–1.095)	0.092
No	136 (62.7)	43 (71.7)	93 (59.2)	1r	
Use of deworming					
Yes	102 (47.0)	26 (43.3)	76 (48.4)	0.815 (0.4481–0.483)	0.503
No	115 (53.0)	34 (56.7)	81 (51.6)	1r	
Use of contraceptive					
Yes	173 (79.7)	48 (80)	125 (79.6)	1.024 (0.487–2.151)	0.950
No	44 (20.3)	12 (20)	32 (21.4)	1r	
Iron-foliate supplementations					
Yes	156 (71.9)	42 (70)	114 (72.6)	0.880 (0.458–1.693)	0.702
No	61 (28.1)	18 (30)	43 (27.4)	1r	
Habit of drinking coffee or tea after meal					
Yes	175 (80.6)	49 (81.7)	126 (80.3)	0.912 (0.425–1.957)	0.814
No	42 (19.4)	11 (18.3)	31 (19.7)	1r	
Consumption of ASF					
Yes	105 (48.4)	21 (35)	84 (53.5)	0.468 (0.253–0.867)	0.016*
No	112 (51.6)	39 (65)	73 (46.5)	1r	
Consumption of PSF					
Yes	35 (16.1)	14 (23.3)	21 (13.4)	1r	0.078
No	182 (83.9)	46 (76.7)	136 (86.6)	0.507 (0.239, 1.079)	
Pregnancy trimester					
1st	73 (33.6)	21 (35)	52 (33.2)	1r	0.350
2nd	66 (30.4)	11 (18.3)	55 (35)	0.495 (0.218–1.127)	
3rd	78 (35.9)	28 (46.7)	50 (31.8)	1.387 (0.698–2.754)	
Parity					
0	132 (61.3)	5 (8.3)	128 (81.5)	1r	0.998
1–4	63 (29)	33 (55)	29 (18.5)	29.131 (10.470–81.054)	
≥5	22 (10.1)	22 (36.7)	0 (0)	4.136E10 (0.000)	
Inter pregnancy interval (years)					
<2	57 (26.3)	46 (76.7)	11 (7)	1r	*<0.0001* *
≥2	108 (49.8)	5 (8.3)	103 (65.6)	0.012 (0.004–0.035)	
Intestinal parasite					
Present	28 (12.9)	21 (35)	7 (4.5)	1r	*<0.0001* *
Absent	189 (87.1)	39 (65)	150 (95.5)	0.087 (0.034–0.219)	

*1r* set as reference, *p* p value, *PSF* plant source food, *ASF* animal source food

* p < 0.05

## Prevalence and severity of anemia

**Fig. 1:**
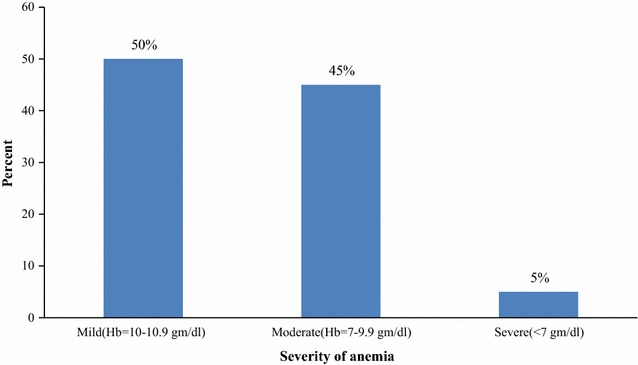
Severity of anemia among pregnant women attending ANC in Butajira General Hospital Southern Ethiopia, March 1–April 30, 2015

Participant’s hemoglobin level was used to determine the prevalence of anemia. The overall burden of anemia among the study participants was 27.6% (60/217). The mean hemoglobin level (adjusted for altitude) was 11.2 gm/dl. From the current occurrence of anemia, 3 (5%) of them were identified as severely anemic, 27 (45%) of them were moderately anemic and 30 (50%) were mildly anemic (Fig. 1).

## Predictors of anemia

Both bivariate and multivariable logistic regression analysis were done to identify the independent predictors of anemia among pregnant women. All the variables were analyzed in bivariate logistic regression analysis and those with a p-value less than 0.05 were considered in multivariable logistic regression analysis. By doing so finally residence, ANC follow up, history of excess menstrual bleeding and interpregnancy interval were identified as predictors of anemia among the study participants (Table 3).

**Table 3:** Multivariable logistic regression analysis of factors associated with anemia among pregnant women (n = 217) in Butajira General Hospital, Southern Ethiopia, March 1–April 30, 2015

Variables	Total (n = 217) n (%)	Anaemia		AOR (95% CI)	p value
		Yes (n = 60) n = (%)	No (n = 157) n = (%)		
Residence					
Urban	168 (77.4)	40 (18.4)	128 (58.9)	0.167 (0.041–0.682)	*0.013* *
Rural	49 (22.6)	20 (9.2)	29 (13.3)	1r	
History of Malaria prior to data collection time					
Yes	54 (24.9)	24 (11)	30 (13.8)	1r	0.525
No	163 (75.1)	36 (16.5)	127 (58.5)	1.622 (0.365–7.211)	
History of excess menstrual bleeding					
Yes	53 (24.4)	16 (7.3)	9 (4.1)	1r	<*0.001* *
No	164 (75.6)	44 (20.2)	148 (68.2)	0.028 (0.006–0.135)	
ANC follow up					
Yes	171 (78.8)	24 (9.2)	147 (67.7)	0.082 (0.018–0.370)	<*0.001* *
No	46 (21.2)	36 (16.5)	10 (4.6)	1r	
Consumption of AFS					
Yes	105 (48.4)	21 (9.6)	84 (38.7)	1.264 (0.365–4.380)	0.712
No	112 (51.6)	39 (17.9)	73 (33.6)	1r	
Inter pregnancy interval (years)					
<2	57 (26.3)	46 (21.1)	11 (5)	1r	*0.016* *
≥2	108 (49.8)	5 (2.3)	103 (47.4)	0.133 (0.026–0.685)	
Intestinal parasite					
Present	28 (12.9)	21 (9.6)	7 (3.2)	1r	0.121
Absent	189 (87.1)	39 (17.9)	150 (69.1)	0.245 (0.042–1.450)	

*1r* set as reference, *AFS* animal food sources

* p < 0.05

## Discussions

Anemia is a worldwide problem affecting all physiologic group. Pregnant women and children are the most vulnerable group for anemia. In the current study, the overall burden of anemia among pregnant women was 27.6%. The burden of anemia in the current study is in harmony with the study conducted by Ethiopian DHS 2005 (27.4%) and a cross-sectional study carried out in Southeast Ethiopia (27.9%) [18, 19]. However, our finding is slightly higher as compared to the finding from central Ethiopia, Addis Ababa (21.3%) [20], and from Northwest Ethiopia (21.6%) [21]. The burden of anemia in the current study is considerably lower than previous study reports from West Arsi Zone, Eastern part of Ethiopia (36.6%) [22], and Jimma Zone, Southwest part of Ethiopia (38.2-53.9%) [1, 23]. Such magnitude differences are due to geographical, economical, seasonal, dietary and behavioral variations [3, 20]. The presences and absences of malaria and intestinal parasitic infection are also among the contributing factors affecting the magnitude of anemia [24]. From multivariable logistic regression analysis, a significant association was found between anemia and residence, history of excess menstrual bleeding, ANC follow up, and interpregnancy interval. Anaemia among pregnant women who live in an urban setting is 0.167 times as likely as anemia among pregnant women who live rural setting. Association of rural residence with the occurrence of anemia has also been reported earlier [1, 19, 23]. The reason for a higher burden of anemia among pregnant women from rural areas may be related to inaccessibility of health care centers. Thus, they lack information on factors causing anemia and possible strategies to prevent the risk factors of anemia. Pregnant women with a history of heavy or excess menstrual cycle were more anemic than those with normal menstruation cycle [19, 23]. In the present finding, the odds of anemia among pregnant women with the normal menstrual cycle is 0.028 times the odds of anemia among pregnant women who had heavy or excess menstrual cycle. History of excessive bleeding can lead to anemia. The anemia may make her feel weak and tired, and she may also experience shortness of breath, rapid heart rate, and lightheadedness. The odds of anemia among pregnant women who had ANC follow up were 0.918 times less likely than anemia among pregnant women who didn’t have ANC follow up. This result is inconsistent with the study conducted in Addis Ababa [20]. Pregnant women who attend ANC follow up were supported by health professionals to prevent anemia. Antenatal care counseling helps in motivating pregnant women to take iron rich food and iron-folic acid tablets. The present study has shown a statistically significant association between interpregnancy interval and anemia. The odds of anemia among pregnant women who had a birth interval of greater than or equal to 2 years were 0.867 times lower than the odds of anemia among pregnant women who had less than 2 years’ birth interval. Appropriate time after each pregnancy for recovery and replenishment of nutrient stores requires 2–5 years. Pregnancy with a short birth interval leads to iron deficiency anemia as iron requirements are substantially higher than the average [25]. Similar finding has also been documented in the studies conducted at Addis Ababa [20], Jimma Hospital [23], and Nigeria [26]. In this particular study, malaria and intestinal parasitic infection, parity, trimester, age, educational status, occupation and income did not show significant association with anemia among pregnant women but found to be significant by other studies [14, 19–22, 27].

## Limitation of the study

Because of the limited fund we had, a number of potential confounding factors such as vitamin B_12_ and/or folate levels, hemoglobinopathies, HIV/AIDS infection, and food frequency, along with a C-reactive protein test for inflammation, were not measured.

## Conclusions

The prevalence of anemia among pregnant women in this study was 27.6%. Living in a rural residence, having a history of excess bleeding during the menstrual cycle, ANC follow up and interpregnancy interval were identified as predictors of anemia among the study participants. Therefore, working in the identified gaps could reduce the current burden of anemia among pregnant women in the study area.

## Abbreviations

AFS: animal foods sources
ANC: Antenatal Care Clinic
EDHS: Ethiopian Demographic Health Survey
Hb: hemoglobin
MCV: mean cell volume
MCH: mean cell hemoglobin
MCHC: mean cell hemoglobin concentration
SNNPR: South Nation Nationality and Peoples Region
SOPs: standard operating procedures
SPSS: statistical packages for social sciences
WHO: World Health Organization
